# P-1831. Hepatitis C Virus (HCV) treatment during pregnancy using glecaprevir/pibrentasvir

**DOI:** 10.1093/ofid/ofaf695.2000

**Published:** 2026-01-11

**Authors:** L Madeline McCrary, Megan R Curtis, Jeannie C Kelly, Patricia Werner, Jessica R Elrod-Gallegos, Tracey Habrock-Bach, Laura R Marks

**Affiliations:** Washington University School of Medicine, St. Louis, MO; Washington University School of Medicine, St. Louis, MO; Washington University School of Medicine, St. Louis, MO; Washington University School of Medicine, St. Louis, MO; Washington University School of Medicine, St. Louis, MO; Washington University School of Medicine, St. Louis, MO; Washington University in St. Louis, St Louis, Missouri

## Abstract

**Background:**

Hepatitis C Virus (HCV) prevalence is increasing among women of reproductive age. HCV infection during pregnancy is associated with adverse outcomes, including preterm birth and perinatal transmission. While effective and well-tolerated direct acting antivirals (DAAs) are available, those diagnosed with HCV during pregnancy are rarely linked to treatment postpartum. Current Infectious Diseases Society of America and American Association for the Study of Liver Disease (IDSA/AASLD) HCV guidelines endorse HCV treatment during pregnancy through shared decision making.

**Figure:**
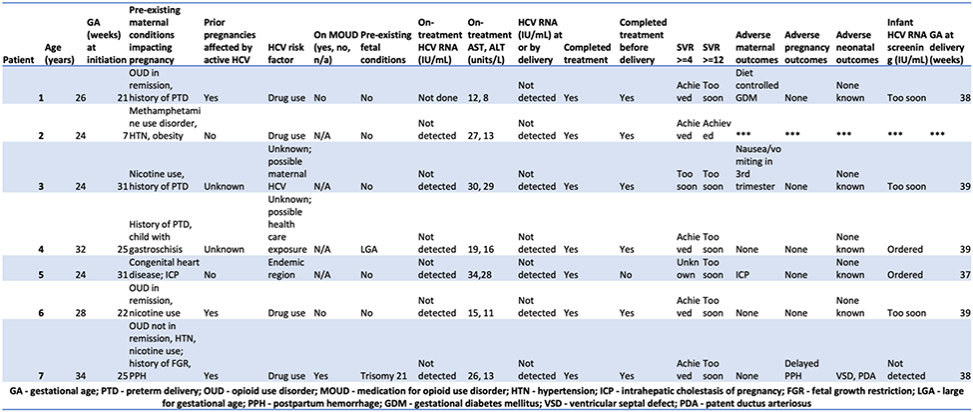
Maternal, fetal characteristics and HCV treatment-related outcomes

**Methods:**

We conducted a retrospective review of pregnant patients with HCV who completed antepartum treatment with glecaprevir/pibrentasvir (GP) between March 2024 and April 2025. Patients were included if they had completed treatment or achieved sustained virologic response (SVR) by the time of this report. Maternal baseline characteristics, and HCV-related, pregnancy and infant outcomes were collected.

**Results:**

Seven patients received antepartum HCV treatment (Table 1). One patient started GP in the first trimester before recognition of pregnancy and elected to continue treatment; the remaining patients initiated GP after 20 weeks’ gestation through shared decision making. All patients with HCV RNA testing during treatment had undetectable viral loads. No elevations in AST or ALT were observed during therapy. All patients had undetectable HCV RNA at or by the time of delivery. SVR testing at or beyond 4 weeks post-treatment (SVR>4) was available in 5/7 patients, all achieved SVR. Among the 6 patients who delivered by the time of this report, no adverse outcomes attributable to GP were identified and all delivered at term. 3 infants were eligible for exposed infant screening; 1 had an undetectable HCV RNA at 4 months old and 2 had testing ordered.

**Conclusion:**

This is the largest cohort of patients with HCV treated with GP during pregnancy. There were no liver enzyme abnormalities while on treatment. No adverse pregnancy or neonatal outcomes attributable to GP were identified. Among the 5 patients with SVR>4 data, all achieved SVR. This report adds to growing evidence that glecaprevir/pibrentasvir can be considered for antepartum use through shared decision making.

**Disclosures:**

L. Madeline McCrary, MD, Gilead FOCUS Program: Grant/Research Support Tracey Habrock-Bach, BS, Gilead FOCUS: Grant/Research Support Laura R. Marks, MD, PhD, Gilead FOCUS Program: Grant/Research Support

